# Breed-Specific Responses and Ruminal Microbiome Shifts in Dairy Cows Under Heat Stress

**DOI:** 10.3390/ani15060817

**Published:** 2025-03-13

**Authors:** Zichen Wang, Mengling Guo, Yan Liang, Fuzhen Zhou, Huiming Zhang, Mingxun Li, Zhangping Yang, Niel Karrow, Yongjiang Mao

**Affiliations:** 1 Key Laboratory for Animal Genetics, Breeding, Reproduction and Molecular Design of Jiangsu Province, College of Animal Science and Technology, Yangzhou University, Yangzhou 225009, China; shirleywang2020@126.com (Z.W.); g18852720440@163.com (M.G.); mz120181016@yzu.edu.cn (Y.L.); zfzstrive@163.com (F.Z.); minmin-911@163.com (H.Z.); limingxun@live.com (M.L.); yzp@yzu.edu.cn (Z.Y.); 2 Center for Genetic Improvement of Livestock, Department of Animal Biosciences, University of Guelph, Guelph, ON N1G 2W1, Canada; nkarrow@uoguelph.ca

**Keywords:** heat stress, Holstein, Jersey, bacteria, fungi

## Abstract

Holstein and Jersey cows, as excellent dairy breeds, have their own advantages in milk yield, milk quality, disease resistance, and heat resistance. However, the adaptability and rumen microbiome changes in Holstein and Jersey cows under heat stress are not clear. Therefore, the main objective of this study was to compare the differences in heat tolerance and the changes in the ruminal microbiome in Holstein and Jersey cows under heat stress. The experiment comprised a 7-day thermo-neutral (TN) period and a 7-day heat stress (HS) period. Five Jersey cows and five Holstein cows with similar parity and days in milk were selected, and rumen fluid was collected from five of them each. Compared with the TN period, heat stress increased the respiratory rate (*p* < 0.05), whereas decreased the milk yield (*p* < 0.01) in the Holstein and Jersey cows. Also, heat stress increased the rectal temperature (*p* < 0.01) in the Holstein cows. Jersey cows had a significantly (*p* < 0.05) lower level of acetic acid, propionic acid, butyric acid, valeric acid, and TVFA during HS compared with the TN period. Furthermore, high-throughput sequencing revealed that the relative abundance of Bacteroidetes and Prevotella increased while the relative abundance of Firmicutes decreased in Holstein cows during the HS period, whereas Christensenellaceae and Clostridium were more abundant in Jersey cows during the HS period than in the TN period. Simultaneously, the dominant fungi in Holstein cows were Ascomycota, Neocallimastigomycota, and Aspergillus. Correlation analysis also provided a link between the significantly altered rumen microbiota and animal production. These results suggest that heat stress has negatively influenced the physiological parameters, milk production, and rumen microbiota of Holstein and Jersey cows. Changes in the rumen fermentation and ruminal microbiome in Holstein cows may be associated with a better adaptation ability to heat stress. Our findings may inform future research to better understand how heat stress affects the physiology and productivity of dairy cattle breeding in southern China and the development of mitigation strategies.

## Simple Summary

Heat stress has a negative effect on milk yield, reproductive performance, and disease resistance of dairy cows, which causes huge economic losses to the dairy industry. Heat stress has become an important factor restricting the development of the dairy industry. This study showed that the adaptability of Holstein and Jersey cows to the thermal environment was associated with changes in rumen bacteria and fungi. Our findings provide new insight into the effects of heat stress on different breeds and provide a scientific basis for dairy cattle breeding in southern China.

## 1. Introduction

Heat stress, as a major environmental stressor, is a worldwide problem for dairy production [1]. Dairy cows are particularly sensitive to high ambient temperatures due to a lack of sweat glands [2]. Most dairy farms in southern China are in hot and humid environments during the summer; thus, cows are not well adapted to the excessive thermal stress. According to statistics, there were 129,000 dairy cattle herds in Jiangsu Province by the end of 2020 [3]. Jiangsu Province is located in the climatic transition zone between the subtropical and warm temperate zones, with an average summer duration of 104 days. Extreme temperatures typically occur in July or August, during mid-summer [3]. During heat stress, thermal balance cannot be maintained when animals are unable to dissipate sufficient metabolic heat or absorb heat [4]. To better determine the influence of heat stress on dairy animals, multiple studies provide evidence that when the temperature–humidity index (THI) thresholds are exceeded, the respiratory rate and rectal temperature begin to increase [1]. Subsequently, heat stress negatively affects nutrition levels, milk production, and reproductive performance, all of which lead to economic losses [5].

Notably, the various breeds have different responses to heat stress in their own way. For example, Angus cattle exhibit higher respiration rates, core body temperatures, and less thermotolerance than Romosinuano cattle [6]. Holstein cows, as the largest source of raw milk, originated from the Netherlands. Jersey is another widely used dairy cattle breed [7]. It has already been pointed out that the antioxidant capacity of Jersey cows is better than that of Holstein cows [8]. The most prominent feature of Jersey cows is a higher milk fat content compared with Holstein cows. Jersey cows have better disease resistance and heat resistance. High milk production and basal metabolism increase the body heat load and thus amplify heat strain in Holstein cows [9].

As a unique organ of ruminants, the rumen is a huge fermentation compartment in which microorganisms digest nutrients, thereby eventually converting feed into milk and meat [10]. The rumen microbiota consists of bacteria, fungi, protozoa, and archaea, which play an important role in the immunity and metabolism of ruminants [11]. With the advent of high-throughput sequencing technologies, the species composition and diversity of microorganisms can be rapidly identified by PCR amplification of hypervariable regions such as 16S, 18S, and the internal transcribed spacer (ITS) of microorganisms [12]. 16S rRNA gene sequencing determines specific regions (V3-V4) of the target gene, which can reflect changes in bacterial diversity [13]. ITS is a non-coding rDNA spacer region located between the 18S and 28S rRNA genes [14]. Hence, the rumen microbiome has deepened our understanding of the major microbial populations and functional pathways associated with responses to increased feed use efficiency, decreased methane emissions, mastitis, and ketosis [15].

The effects of heat stress on microbial composition in cows have been studied in recent years and it has been found that heat stress breaks the structural balance of the rumen microbiota [16]. For example, Holstein cows were shown to have an enrichment of Fibrobacillus in the rumen under heat stress, which may be a source of heat generation [17]. It is worth noting that rumen homeostasis is the basis of immunity, metabolism, development, and health in ruminants [18]. To our knowledge, although there have been comparative studies on physiological responses, there are a limited number of studies regarding the differences in rumen microorganisms in different dairy breeds under heat stress [18]. The study of T Park et al. [19] showed that heat stress had varying degrees of effects on the prokaryotes, fungi, and protozoa of the rumen microbiota of lactating Holstein cows. F Ceciliani et al. [20] showed that the Brown Swiss cow milk microbiota was less affected by HS than the Holstein cow milk microbiota. We hypothesized that specific changes in the rumen microbiome of different breeds of dairy cows under heat stress might influence animal performance. Consequently, the present study compares the adverse effects of heat stress on physiological, production, rumen parameters, and rumen microbiome between Holstein and Jersey cows throughout the 16S rRNA gene and ITS sequencing.

## 2. Materials and Methods

### 2.1. Animals and Diets

This trial was carried out at a dairy farm in Jiangsu Province in August 2023 for a heat stress (HS) period and in May 2023 for a thermo-neutral (TN) period for 7 days, respectively. Five Holstein cows averaging days in milk (DIM) of 51.6 ± 4.80 and five Jersey cows averaging DIM of 42.2 ± 1.50 in the TN period were used in this study. Finally, five healthy Holstein cows averaging a parity of 1.59 ± 0.12, a body weight of 611.96 ± 37.82 kg, and an age of 36 ± 4.98 months were selected, and five healthy Jersey cows averaging a parity of 1.68 ± 0.11, a body weight of 534 ± 18.22 kg, and an age of 37.73 ± 5.23 months were selected in the TN period. All procedures were approved by the Animal Care and Use Committee of Yangzhou University (approval number: 2006-398).

Five cows from one group were housed in a cowshed with an open sided. Then, the cows were kept in individual pens with free access to water. Shading nets were used around the cowshed and each pen contained a fan during the HS period. Both feeding and milking were performed twice daily and the cows were fed with a total mixed ration (TMR) at a rate of 10% refusal. Samples of feed stuffs and TMR were collected during the TN and HS period, respectively. Feed ingredients and composition were determined according to Association of Official Analytical Chemists (AOAC) standards in the laboratory [21]. The detailed composition and nutrient level of the diets are shown in Table 1.

### 2.2. Sample Collection and Measurement

The automatic temperature and humidity recorder was placed 1.5 m above the ground in the cowshed, avoiding direct sunlight and cowshed spraying, and automatically recorded every 30 min during the test by RC-4HC (Jiangsu Jingchuang Electric Co., Ltd., Xuzhou, China). The data collected consisted of ambient temperature and relative humidity. From these data, the THI was calculated using the following equation (NRC 1971): THI = 0.81 × T + (0.99 × T − 14.3) × R + 46.3, where T is the ambient temperature (°C); R is the relative humidity (%). Respiratory rate and rectal temperature were measured at 1400 during each experimental period. Briefly, rectal thermometry was determined using an electronic thermometer inserted into the rectum to a depth of 7 cm and the number of flank movements of the cow within 1 min as the respiratory rate [18].

Milk yield was recorded for each cow twice daily, and an individual milk sample was collected from each cow at morning milking. Then, the milk composition of milk samples, including milk fat percentage, milk protein percentage, and lactose percentage, were analyzed by a MilkoScan FT-1 multifunctional dairy analyzer (Foss Electric, Hilleroed, Denmark) at Jiangsu Dairy Herd Improvement (DHI) Center in Nanjing Agricultural University.

On the last day of each experimental period, an oral stomach tube was inserted to a depth of 20 cm to obtain representative rumen fluid samples as described by Shen JS et al. [19]. After measuring the pH value, samples were collected in duplicate, one for rumen fermentation analysis (0.2 mL of 20% metaphosphoric acid was added and kept at −20 °C) and another one stored at −80 °C until sequencing analysis [20]. To analyze total volatile fatty acid (TVFA) concentration, rumen fluid was centrifuged (10,000× *g* at 4 °C for 10 min) and 1 mL of the supernatant was taken. Subsequently, TVFA was determined by a 7890-B gas chromatograph (GC, Agilent, Santa Clara, CA, USA), following the procedure mentioned by Seankamsorn A et al. [21].

### 2.3. DNA Extraction and MiSeq Sequencing

Following rumen fluid melting on ice, the DNeasy PowerSoil Kit (QIAGEN, Dusseldorf, Germany) was used to extract genomic DNA according to the manufacturer’s instructions. The concentration of DNA was detected by a K5800 microspectrophotometer (KAIAO, Beijing, China) and verified with NanoDrop and 1% agarose gel electrophoresis [20]. For bacteria diversity analysis, the v3–v4 region of the 16S rRNA gene was amplified using primers 343F/798R. For the fungal concentration, primers ITS1 F/ITS2 R were used to target the ITS I variable regions [22]. Amplicon quality was visualized using gel electrophoresis, and purified by using the Tks Gflex DNA Polymerase (Takara Bio, Kusatsu, Japan), against Silva database Version 138 (16s/18s rDNA) using the RDP classifier quantified using the Qubit dsDNA assay kit (Thermo Fisher Scientific, Waltham, MA, USA). Equal amounts of purified amplicon were pooled for building libraries for sequencing on the Illumina MiSeq platform according to the standard protocol.

### 2.4. Sequence Bioinformatic Analysis

According to the overlapping order, only the sequences with overlapping exceeding 10 bp were assembled. After trimming, paired-end reads were merged as raw tags using FLASH software (v1.2.3). Parameters of the assembly included 20% of maximum mismatch rate, and 10 bp of minimal overlapping, while 200 bp of maximum overlapping were removed [23]. Meanwhile, the chimera sequences were identified and removed using UCHIME. After the sequencing data were preprocessed to generate high-quality sequences, parameters with sequence similarity greater than or equal to 99% were classified as operational taxonomic units (OTUs) using Vsearch software(v 2.4.2). The representative 16S rRNA gene sequences of the OTUs were annotated and blasted against the Silva database (Version 138) using the RDP classifier. The ITS sequences were aligned using the Unite database and taxonomically assigned using BLAST (v 2.14.0). Alpha diversity and beta diversity were calculated by QIIME. The vegan and ggplot2 packages in R were used to perform principal component analysis (PCA). Differences in microbiota abundance were analyzed by the linear discriminant analysis effect size (LEfSe) tool.

### 2.5. Data Analysis

Physiological parameters, animal performance, rumen fermentation, and alpha diversity were tested for normality using Shapiro–Wilk’s test and the data conformed to a normal distribution, except for somatic cell score (SCC). SCC was into somatic cell scores (SCS) that were nearly normally distributed by the formula SCS = Log_2_ (SCC/100) + 3 [24]. Then, analyses were performed via the two-way ANOVA procedure of SPSS version 25.00 (IBM Inc., Chicago, IL, USA). The model was as follows:

$$Y_{im}=\mu +P_{i}+S_{m}+P_{i}\times S_{m}+e_{im}$$

In the above model, *Y_im_* is the observed value of physiological parameters, animal performance, rumen fermentation, and alpha diversity; *μ* is the overall mean; *P_i_* is the fixed effect of breed (Holstein and Jersey cows); *S_m_* is the fixed effect of test stage (HS and TN period); $e_{im}$ is the random residual. Differences were considered statistically significant at *p* < 0.05 and the results of these analyses were expressed as means and SEM. The correlations between differential bacteria and fungi in the rumen and physiological parameters, animal performance, and rumen fermentation parameters were analyzed by Person correlation using SPSS version 25.00 (IBM Inc., Chicago, IL, USA), and additionally visualized using GraphPad Prism 7.0 software (GraphPad Software, Inc., La Jolla, CA, USA).

## 3. Results

### 3.1. Heat Stress Response Differences Between Holstein and Jersey Cows

**Figure 1:**
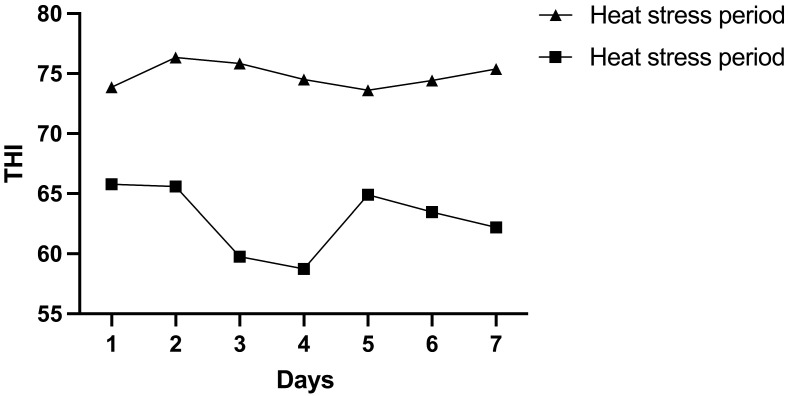
Temperature, humidity, and temperature–humidity index (THI) values of the cowshed during the thermo-neutral period and the heat stress period.

We first calculated the THI value to determine whether animals were under continuous heat stress during the August trial. The overall average daily THI was 74.86 ± 0.79 in August, whereas it was 62.92 ± 2.81 in May (Figure 1). Heat stress had a significant negative effect on physiological parameters, milk yield, and milk quality regardless of breed (Table 2). Both Holstein and Jersey cows had higher respiratory rates (*p* < 0.05) in HS compared with the TN period, whereas milk yield was decreased in HS compared with the TN period (*p* < 0.01). The rectal temperature of Holstein cows during the HS period was higher than that of Jersey cows and also higher than that of Holstein cows during the TN period. However, the milk protein percentage and milk fat percentage of Holstein and Jersey cows were unaffected by HS. Moreover, the milk protein percentage (*p* < 0.01) and milk fat percentage (*p* < 0.05) were greater in the Jersey cows compared with the Holstein cows during the HS period.

### 3.2. Differences in Rumen Fermentation Parameters Between Holstein and Jersey Cows

Volatile fatty acid and pH data were measured to provide the environment state of the rumen at the time when the ruminal digest was collected for sequencing analysis. Rumen fermentation parameters are listed in Table 3. Compared with the Holstein cows under heat stress, Jersey cows had significantly (*p* < 0.05) lower values of acetic acid, propionic acid, butyric acid, valeric acid, and TVFA. It was also found that only Jersey cows had a significantly (*p* < 0.05) lower level of acetic acid, propionic acid, butyric acid, valeric acid, and TVFA during HS compared with the TN period.

### 3.3. Heat Stress Impacts Microbiota Diversity

In this study, 16S rRNA gene and ITS high-throughput sequencing were applied to reveal the differences in the rumen microbiome of Holstein and Jersey cows between the HS and TN period. The 16S rRNA gene revealed that a total of 68,531 clean tags were obtained from ruminal samples, with an average OTU count of 3638 per sample. Similarly, there were a total of 52,436 clean tags detected via the ITS sequencing set, with a mean OTU count of 518 per sample. By determining the alpha diversity within samples, we observed that goods coverage was >96% for bacteria and 99% for fungi, indicating a good sequencing depth for the analysis of the rumen microbiota and it can accurately reflect the composition and diversity of microorganisms in the sample (Table 4). As shown in Table 4, for bacteria, it was observed that Chao1, Shannon, and observed species of Holstein and Jersey cows were lower in HS than in the TN period (*p* < 0.01), but Simpson evenness showed no difference between the HS and TN periods.

With respect to fungi, the Chao 1 and Simpson of the Jersey cows decreased in the HS period compared with the TN period (*p* < 0.05). On the contrary, the Simpson and Shannon of Holstein cows increased in the HT period compared with the TN period (*p* < 0.01). Additionally, Chao1, Simpson, Shannon, and observed species changed during the TN period between the Jersey and Holstein cows throughout ITS sequencing (*p* < 0.01).

**Figure 2:**
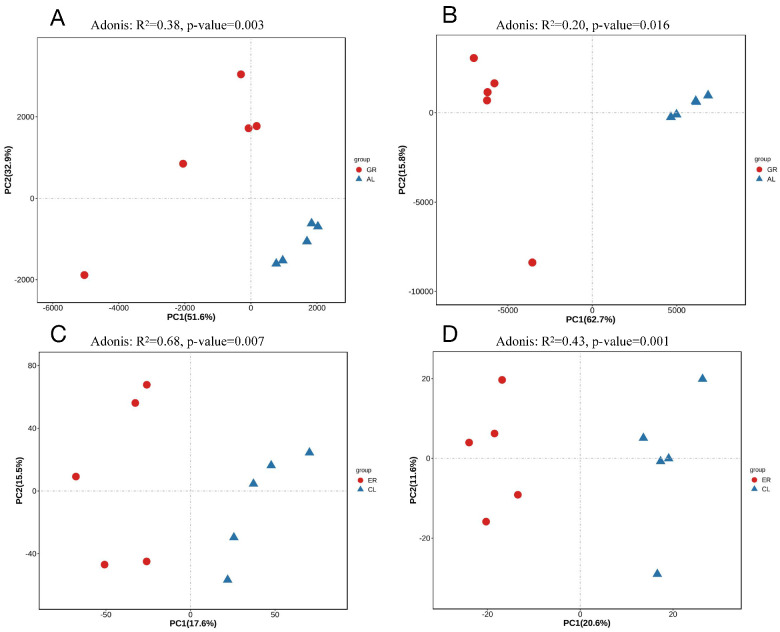
Beta diversity analysis of ruminal bacteria (Holstein, (**A**); Jersey, (**B**)) and fungi (Holstein, (**C**); Jersey, (**D**)) through principal component analysis based on Bray–Curtis. ER—samples of Jersey cows under thermo-neutral; CL—samples of Jersey cows under heat stress; GR—samples of Holstein cows under thermo-neutral; AL—samples of Holstein cows under heat stress.

As depicted in Figure 2, the β-diversity of the bacterial and fungal microbiotas was studied to explore differences in heat stress between Holstein and Jersey cows. In brief, the PCA plot showed an obvious separation between the bacteria from the HS and TN period, same as the PCA plot based on fungi. Overall, the composition of ruminal microbiota was influenced by heat stress.

### 3.4. The Effect of Heat Stress on the Ruminal Microbiota Communities in Holstein and Jersey Cows

**Figure 3:**
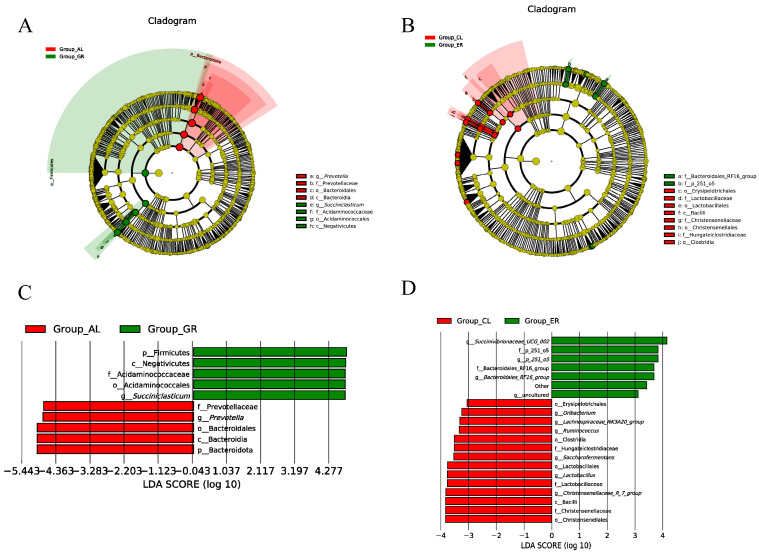
The linear discriminant analysis effect size (LEfSe) plots of differentially abundant ruminal bacterial taxa by heat stress. Cladogram of differential bacteria from phylum to genus in Holstein (**A**) and Jersey (**B**); red or green nodes represent the microbe that has a significant difference between the two groups. Taxonomic comparison of ruminal bacteria in Holstein (**C**) and Jersey cows (**D**). Linear discrimination analysis (LDA) score is used to determine the effect size. ER—samples of Jersey cows under thermo-neutral; CL—samples of Jersey cows under heat stress; GR—samples of Holstein cows under thermo-neutral; AL—samples of Holstein cows under heat stress.

**Figure 4:**
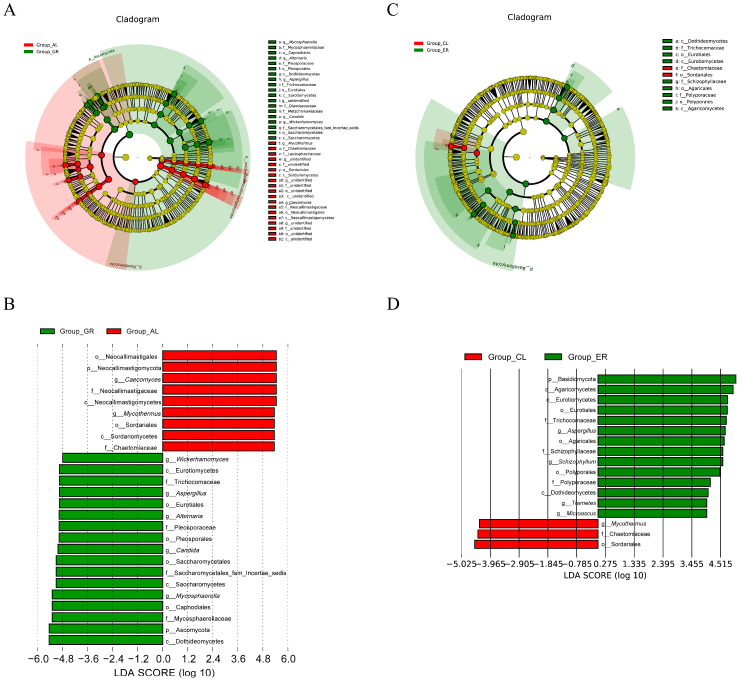
The linear discriminant analysis effect size (LEfSe) plots of differentially abundant ruminal fungi taxa by heat stress. Cladogram of differential fungi from phylum to genus in Holstein (**A**) and Jersey (**C**); red or green nodes represents the fungi that have a significant difference between the two groups. Taxonomic comparison of ruminal fungi in Holstein (**B**) and Jersey cows (**D**). Linear discrimination analysis (LDA) score is used to determine the effect size. ER—samples of Jersey cows under thermo-neutral; CL—samples of Jersey cows under heat stress; GR—samples of Holstein cows under thermo-neutral; AL—samples of Holstein cows under heat stress.

To further determine the ruminal microbiome composition that was responsible for the shift due to HS in Holstein and Jersey cows, bacterial and fungal taxa with a relative abundance of >1% were subjected to taxonomic composition analysis. The significantly different abundant ruminal bacteria and fungi were identified by LEfSe analysis. As shown in Figure 3, there was an increased relative abundance of Bacteroidota, Bacteroidales, and Prevotella in Holstein cows during HS compared with the TN period, whereas there was a decreased relative abundance of Firmicutes, *Succiniclasticum*, and Acidaminococcaceae in Holstein cows during HS compared with the TN period. Especially, the relative abundance ratios of Firmicutes-to-Bacteroidetes in Holstein and Jersey cows under heat stress were 0.25 (0.74/0.118) and 0.36 (0.68/0.24), respectively. Moreover, the relative abundance of *Lachnospiraceae_NK3A20_group*, *Ruminococcus*, Clostridia, Lactobacillus, *Christensenellaceae_R_7_group*, and Christensenellaceae was higher in Jersey cows during HS compared with the TN period, while *Succinivibrionaceae_UCG_002* and *Bacteroidales_RF16_group* at the genus level were more predominant in Jersey cows during TN compared with the HS period. LEfSe analysis of differentially abundant ruminal fungi taxa by heat stress (Figure 4) revealed that, in Holstein cows, Chaetomiaceae, Lasiosphaeriaceae, and Neocallimastigaceae were significantly higher in the HS period than in the TN period. In contrast, there was a decrease in the relative abundance of Mycosphaerellaceae, Pleosporaceae, Trichocomaceae, and Saccharomycetes in Holstein cows during HS compared with the TN period. During analysis of the rumen samples of Jersey cows, most of the fungi species found with elevated during the HS period were Chaetomiaceae and *Mycothermu*, while during the TN period, there was an increase in the relative abundance of Eurotiales and Eurotiales.

### 3.5. Correlation Between Differential Ruminal Microorganisms, Heat Stress Response Indices, and Rumen Fermentation Performance

**Figure 5:**
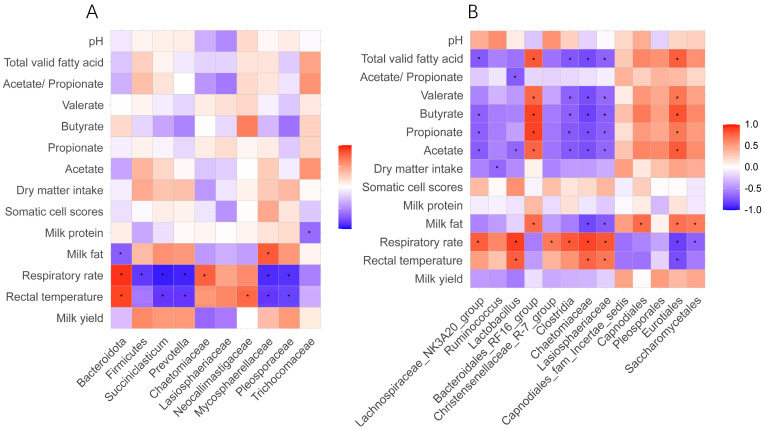
Correlation between differential ruminal microorganisms and heat stress response indices of Holstein cows (**A**) and Jersey cows (**B**) under heat stress.

For Holstein cows (Figure 5A), significantly positive correlations were found between respiratory rate and rectal temperature with Bacteroidota (r < 0.5, *p* < 0.05). The respiratory rate was negatively correlated (r > 0.5, *p* < 0.05) with three bacteria and two fungi. Also, the rectal temperature was negatively correlated (r > 0.5, *p* < 0.05) with two bacteria and two fungi. With respect to Jersey cows (Figure 5B), VFA including butyrate, propionate, and acetate were negatively correlated (r > 0.5, *p* < 0.05) with *Lachnospiraceae_NK3A20_group*, Clostridia, Chaetomiaceae, and Lasiosphaeriaceae. In addition, the respiratory rate was negatively correlated (r > 0.5, *p* < 0.05) with three bacteria and two fungi but positively correlated (r < 0.5, *p* < 0.05) with four bacteria and two fungi. Also, the rectal temperature was negatively correlated (r > 0.5, *p* < 0.05) with two bacteria and two fungi.

## 4. Discussion

Both ambient temperature and humidity are critical factors in assessing the comfort of dairy cows. These parameters are summarized by THI, a widespread measure indicating the margins of the thermal neutrality for dairy cows [22]. Cows in this study that were exposed to a THI > 68 showed that the heat load during HS was higher than during the TN period. Respiratory rate and rectal temperature are stress indicators that are clearly evident and could be altered by thermal stress [23]. As expected, our experiments observed a significant increase in rectal temperature and respiratory rate in both Jersey and Holstein cows during the HS period, which was similar to the results by S I Ul Umar et al. [24] and A Kumar et al. [25]. Also, S Tao et al. [26] reported that Holstein cows had a rectal temperature of 39.5 °C and a respiratory rate of 70 breaths per minute. The increase in respiratory rate is for heat dissipation, as 65% of body heat is lost through the respiratory tract, thus losing heat to the environment [27]. The rectal temperature of Holstein cows during the HS period was significantly higher than that of Jersey cows, indicating that Jersey cows have stronger thermoregulatory ability and better thermal adaptability under high-temperature environments. Such changes in physiological parameters are adaptive mechanisms initiated by lactating cows to restore their thermal balance [28].

Heat stress also leads to reduced performance due to the increased energy required to drive heat loss [29]. Many studies have reported the relationship between THI and the amount of milk yield change. For each unit increase in THI, Holstein cows decreased 0.88 kg/day milk yield [4]. Moreover, there was a decrease in milk production of 21% in cows when THI exceeded 78 [30]. In previous studies, it has been reported that Jersey cows have greater heat tolerance than the Holstein breed for milk production [8]. Evaluating the effect of HS by comparing seasons, HS decreased the milk yield in Jersey and Holstein cows in our study, whereas there was no difference in milk yield between Jersey and Holstein cows. Furthermore, milk quality in the current study was affected by breed, with the milk fat percentage and milk protein percentage of Jersey cows being higher than those of Holstein cows. These differences may not be due to the response to HS, rather only milk quality differences between breeds. This is similar to the results of others who noted that Jersey cows have the highest milk quality in the world [31]. In addition, heat stress altered the milk composition of the Jersey cows compared with the TN period. Consequently, the mammary synthetic capacity was likely impaired by heat stress in the Jersey cows. This also indicated that the nutritional level of feed under heat stress may not meet the nutritional needs of dairy cows, especially Jersey cows, after we restricted the feeding of the Jersey and Holstein cows. In the studies evaluating the effect of HS by comparing seasons, somatic cell counts were found to increase, which could be caused by other stress conditions such as a sudden change in the milking regimen or social isolation [32]. These variations in somatic cell count were not present in our study. Not entirely a result of temperature, changes in SCC may also be associated with increased pathogens in the pen environment [33]. Collectively, these findings indicate that, regardless of breed, cows are negatively affected by heat stress in terms of milk yield and milk quality.

Rumen TVFA concentration can be used as an indicator of energy balance and energy utilization in dairy cows, while increased rumen fermentation may generate an additional heat load in dairy cows [34,35]. In this study, the difference in volatile fatty acid content between Jersey and Holstein cows under heat stress may be partly responsible for the higher milk fat and milk protein content in Jersey compared with Holstein cows. Previous studies [36] have revealed that TVFA varies in different species, e.g., compared with the high concentrate ratio in the dietary composition of dairy cows, buffaloes are more resistant to coarse feeding, which may affect the low TVFA content of buffaloes compared with dairy cows. The finding also demonstrated the decrease in TVFA concentration in Jersey cows under heat stress. The decrease in TVFA may lead to a decrease in milk yield and milk quality [34]. This observation may be supported by the fact that the content of propionate and butyric acid may be used to meet energy needs and milk fat synthesis [37]. Propionic acid is used for glucose synthesis in the liver and butyric acid mainly provides the energy demand of the rumen epithelium [38]. In addition, acetic acid and propionic acid are precursors of fatty acids and cholesterol, which are related to milk fat content [36]. Propionic acid is also involved in the synthesis of lactose and milk protein [39]. The decrease in TVFA may also lead to an increase in digestibility, which is associated with increased dry matter and fiber digestibility, whereas the hydrolysis products are not fermented to VFA [40]. Taken together, the rumen fermentation types of Holstein and Jersey cows are different, and heat stress negatively affects rumen fermentation and milk synthesis in Jersey cows.

We attempted to reduce inter-individual variation by selecting cows with close lactation stages and parities. Also, we used restricted feeding for both breeds and there was no difference in feed intake. Consequently, we assumed that the differences in rumen microbial diversity and abundance observed in lactating cows were mainly attributed to the effect of ambient temperature and humidity. Overall, HS reduced the species richness of bacteria in Jersey and Holstein cows, which is similar to the results of a previous study indicating that HS altered the rumen microbial population in dairy cows [41]. Firmicutes and Bacteroidetes, as the two predominant phyla in ruminal bacteria, play an important role in carbohydrate degradation [42]. Our results showed a greater abundance of Bacteroidetes in Holstein cows and a lower abundance of Firmicutes in Holstein cows during the HS period compared with the TN period. Also, the Firmicutes-to-Bacteroidetes ratio in Holstein cows was lower than in Jersey cows under heat stress. Of note, some studies have suggested that the ratio of Firmicutes to Bacteroides is positively correlated with feed efficiency, milk yield, and milk fat yield [43]. Corresponding to this, the ratio of Firmicutes/Bacteroides decreased in Holstein cows during the summer as they may not obtain more energy from the diet to meet their maintenance and production needs [44]. Prevotella species, which are the major genera in the rumen, were highly abundant in Holstein cows during the HS period compared with the TN period. Prevolla may be the basis for inhibiting methane production [45]. Similarly, Prevotella_9 was found more abundant in the Holstein cows compared with the Jersey cows under the HS period. Therefore, this is advantageous for Holstein cows, because rumen metabolism shifts to unusable final products such as methane have been shown to occur mostly in inefficient cows [46]. Regarding the shift of ruminal bacteria of Jersey cows between the HS and TN periods with classification to the genera level, Christensenellaceae increased in abundance in Jersey cows under heat stress, being associated with immune regulation and health homeostasis and also with feed efficiency [47]. The present study showed that Jersey cows had lower *Lachnospiraceae_NK3A20_group* and Clostridium under TN compared with the HS period. Among them, Clostridium has also been implicated in maintaining gastrointestinal health [48]. According to M Boutard et al. [49], *Lachnospiraceae_NK3A20_group* act as members of the Clostridium order, which are considered to be able to ferment polysaccharides into butyrate and acetate acid in the rumen. Based on these findings, we speculate that the rumen bacterial community differs in diversity between Holstein and Jersey cows, possibly because they have undergone different selection processes in response to heat stress.

Despite fungi being the second largest kingdom of eukaryotic life, there are still a large number of unannotated genes after sequencing due to existing databases such as NCBI or KEGG lacking information on fungi. It is well-known that fungi are the most efficient cellulose-degrading microorganisms, which can synthesize a large number of cellulase and hemicellulase systems and improve roughage utilization [50]. Additionally, fungi can also synthesize xylanase to decompose xylan, while participating in the degradation and synthesis of insoluble proteins [51]. Based on these findings, we speculate that the fungi community may play an important role in dairy ruminal digestive metabolism and energy production. In the present study, the diversity of rumen fungi in Jersey cows during the TN period was higher than that in Holstein cows and significantly higher than that in Jersey cows during the HS period. It is worth noting that the linear discriminant analysis effect size (LEfSe) plot of rumen fungal taxa shows that the dominant phylum of different groups is different. These dominant fungi, including Ascomycota, Neocallimastigomycota, and Aspergillus, are similar to the results of rumen anaerobic fungi in most cow rumen fluid samples [52]. H Wang et al. [53] described Ascomycota as the dominant ruminal fungi in cows, mainly used as a degrader of persistent organic substances such as lignin and keratin in the nutrient cycle. Aspergillus fungi are a widely distributed class of filamentous fungi that degrade cellulose, hemicellulose, and lignin [54]. Neocallimastigomycota possesses an abundance of cellulase and hemicellulase systems that efficiently degrade cellulose and hemicellulose in plant cell walls, convert them into fermentable monosaccharides, and mainly consume rumen-degradable proteins to produce high-quality microbial proteins for the host [55,56]. Based on these findings, we speculate that Neocallimastigomycota can improve the overall metabolic efficiency and nutrient utilization of the host to reduce metabolic calorie production associated with digestion, which is essential during heat stress with elevated metabolic rates. Mycothermus is a thermophilic fungus that thrives in high-temperature environments [57]. This is consistent with our study that Mycothermus was highly abundant in both Holstein and Jersey cows under heat stress. As a thermophilic organism, Mycothermus may produce metabolites that help stabilize cellular structures or enzymes at elevated temperatures [58]. However, studies on Mycothermus and its specific metabolites are limited and mainly focus on plants, and the effects on heat tolerance in dairy cows need to be explored. Taken together, these findings suggest that the environment or breed may affect the performance of dairy cows by influencing feed conversion efficiency.

The findings in this study confirm and extend previous studies by demonstrating polymorphic changes in rumen bacteria and fungi between Holstein and Jersey cows. Changes in the rumen fermentation and ruminal microbiome in Holstein cows may be associated with a better adaptation ability to heat stress. Our findings provide new insight into the effects of heat stress on different breeds and provide a scientific basis for dairy cattle breeding in southern China.

## 5. Conclusions

In conclusion, both Holstein and Jersey cows during the HS period had lower milk yield, higher respiratory rates, and higher rectal temperature than during the TN period. Heat stress altered the relative abundance of Bacteroidetes and Firmicutes in Holstein cows and the relative abundance of Christensenellaceae and *Lachnospiraceae_NK3A20_group* in Jersey cows. The dominant ruminal fungi of Holstein and Jersey cows include Ascomycota, Neocallimastigomycota, and Aspergillus. These bacteria and fungi play an important role in dairy ruminal digestive metabolism and energy production. These findings reveal the negative effects of heat stress on physiological parameters and milk performance in dairy cows and provide polymorphic changes in rumen bacteria and fungi between Holstein and Jersey cows. However, our standing of the mechanism by which the differential rumen microbiome and its host interact is still in its infancy.

## Figures and Tables

**Table 1:** Composition and nutrient levels of the diet fed to Holstein and Jersey cows (DM basis, %).

Ingredients	Content	Nutrient Levels	Content
Corm silage	41.20	NEL (MJ/kg)	6.36
Alfalfa hay	8.24	CP	18.47
Wheat straw	4.14	EE	4.85
Beer slack	20.60	NDF	33.39
Concentrate supplement 1	24.42	ADF	18.97
NaCl	0.55	Ca	0.95
Na_2_CO_3_	0.85	P	0.50
Total	100.00		

Note: (1) Composition of concentrate supplement showed as follows: corm 51.70%, barley 4.00%, soybean meal 22.80%, canola meal 8.00%, corn DDGs 7.50%, fat powder 1.70%, CaHPO_4_ 0.40%, CaCO_3_ 1.50%, MgO 0.60%, NaCl 0.80%, premix 1.00%. Contained the following per kg of the premix: VA ≥ 1500,000 IU, VD3 ≥ 300,000 IU, VE ≥ l,800,000 IU, nicotinic acid ≥ 2500 mg, Cu ≥ l500 mg, Fe ≥ 5600 mg, Mn ≥ 4500 mg, Zn ≥ 9600 mg, I ≥ 150 mg, Se ≥ 40 mg, Co ≥ 60 mg.

**Table 2:** Animal performance of Holstein and Jersey cows under heat stress and during the thermo-neutral period.

Index	Thermo-Neutral			Heat Stress			*p*-Value		
	Holstein Jersey SEM			Holstein	Jersey	SEM	Breed	State	Breed × State
Respiratory rate, bpm	39.70	38.38	3.06	66.13 ^A^	65.42 ^A^	6.29	0.812	0.001	0.692
Rectal temperature, ℃	37.93	37.54	0.16	38.62 ^Aa^	38.12	0.22	0.009	<0.001	0.560
Dry matter intake, kg/d	14.76	11.70	1.33	13.90	11.02	0.84	0.060	0.126	0.609
Milk yield, kg/d	26.06 ^A^	20.00 ^A^	3.11	21.26	16.94	2.15	0.054	0.014	0.651
Protein, %	3.63	4.31 ^Aa^	0.26	3.28	3.99 ^a^	0.32	0.001	0.882	0.749
Fat, %	5.12	7.65 ^a^	0.93	4.81	7.38 ^a^	0.91	0.014	0.007	0.803
SCS	3.70	4.36	1.10	3.51	4.65	0.83	0.230	0.940	0.744

Abbreviation: SCS—somatic cell scores. ^a^ Statistically different comparing Holstein × Jersey within the same group. ^A^ Statistically different comparing heat stress × thermo-neutral within the same breed. Values were expressed as mean ± SEM.

**Table 3:** Effect of heat stress on the rumen fermentation parameters of Holstein and Jersey cows.

Index	Thermo-Neutral			Heat Stress			*p*-Value		
	Holstein Jersey SEM			Holstein	Jersey	SEM	Breed	State	Breed × State
pH	6.72	6.66	0.32	6.59	6.59	0.75	0.925	0.686	0.917
Acetate, mmol/L	55.55	59.84 ^A^	9.37	51.18 ^a^	37.64	5.18	0.402	0.026	0.118
Propionate, mmol/L	15.26	15.17 ^A^	1.97	15.53 ^a^	10.5	1.60	0.064	0.104	0.072
Butyrate, mmol/L	6.69	6.90 ^A^	1.22	8.07 ^a^	3.89	0.80	0.017	0.283	0.009
Valerate, mmol/L	0.76	0.69 ^A^	0.09	0.80 ^a^	0.52	0.12	0.032	0.364	0.170
A/P	3.59	3.97	0.29	3.46	3.65	0.35	0.238	0.364	0.670
TVFA, mmol/L	79.65	83.89 ^A^	12.52	78.33 ^a^	53.72	6.84	0.175	0.044	0.063

Abbreviations: A/P—acetate/propionate; TVFA—total valid fatty acid. ^a^ Statistically different comparing Holstein × Jersey within the same group. ^A^ Statistically different comparing heat stress × thermo-neutral within the same breed. Values are expressed as mean ± SEM.

**Table 4:** Effect of heat stress on the richness and diversity of rumen microbiota in Holstein and Jersey cows.

Index	Thermo-Neutral			Heat Stress			*p*-Value		
	Holstein	Jersey	SEM	Holstein	Jersey	SEM	Breed	State	Breed × State
16S rRNA gene									
Goods coverage	0.96	0.98	0.00	0.98 ^A^	0.98	0.00	0.044	<0.001	0.051
Chao1	8147.61 ^A^	7834.82 ^A^	268.55	4428.32	4490.99	147.84	0.427	<0.001	0.238
Simpson indices	0.99	0.99	0.00	1.00	1.00	0.00	0.240	0.137	0.193
Shannon	10.04 ^A^	10.16 ^A^	0.47	9.37	9.47	0.31	0.564	0.002	0.958
Observed species	6000.60 ^Aa^	5362.12 ^A^	470.72	3555.28	3551.58	200.73	0.024	0.421	0.001
ITS									
Goods coverage	0.99	0.99 ^Aa^	0.00	0.99 ^A^	0.99	0.00	0.004	<0.001	0.180
Chao1	417.17	690.77 ^a^	19.34	501.36	412.41 ^A^	80.60	0.041	0.032	<0.001
Simpson indices	0.84	0.98 ^a^	0.01	0.94 ^A^	0.93 ^A^	0.02	<0.001	0.065	<0.001
Shannon	4.09	7.26 ^Aa^	0.68	5.97 ^A^	5.52	0.60	<0.001	0.727	<0.001
Observed species	343.14	599.44 ^Aa^	136.90	473.70	399.20	127.52	0.043	0.412	0.001

^a^ Statistically different comparing Holstein × Jersey within the same group. ^A^ Statistically different comparing heat stress × thermo-neutral within the same breed. Values are expressed as mean ± SEM.

## Data Availability

The 16S rRNA gene and internal transcribed spacer data are available in the NCBI database under accession PRJNA953595.

## Abbreviations

THI—temperature–humidity index; DIM—days in milk; ITS—internal transcribed spacer; HS—heat stress; TN—thermo-neutral; TVFA—total volatile fatty acid; OTU—operational taxonomic unit; PCA—principal component analysis; LEfSe—linear discriminant analysis effect size; SCC—somatic cell score; SCS—somatic cell scores.
